# Colonic Metastasis in a Patient With Hereditary Diffuse Gastric Cancer: A Case Report

**DOI:** 10.7759/cureus.59483

**Published:** 2024-05-01

**Authors:** Sharon John, Carlos Cantu Lopez, Sarahi Herrera-Gonzalez, Yatinder Bains

**Affiliations:** 1 Internal Medicine, St. George's University School of Medicine, St. George's, GRD; 2 Internal Medicine, Saint Michael's Medical Center, Newark, USA; 3 Gastroenterology and Hepatology, Saint Michael's Medical Center, Newark, USA

**Keywords:** iron deficiency anemia (ida), diffuse hereditary gastric cancer, linitis plastica, colon metastasis, gastric cancer

## Abstract

Metastasis of gastric carcinoma to atypical locations can complicate management, often leading clinicians to rely heavily on chemotherapy. While instances of gastric carcinoma spreading to the liver, peritoneum, and lymphatics are well documented in the literature, there is limited evidence of its spread to intraintestinal organs, particularly the colon. This scarcity of reports complicates diagnosis, given the variations in histopathology. This case report highlights a 35-year-old patient diagnosed with colonic metastasis from hereditary diffuse gastric cancer (HDGC) while being evaluated for potential causes of iron deficiency anemia. A mutation in the E-cadherin (CDH1) tumor suppressor gene is associated with HDGC. Dysregulation of CDH1 leads to tumor proliferation, invasion, migration, and metastasis. Treatment options for gastric cancer include surgical resection with neoadjuvant or adjuvant chemotherapy or palliative care with chemotherapy in metastatic disease. Although colonic metastasis from gastric cancer is rare, documented incidents can offer valuable insights that avoid misdiagnosing primary tumors and help guide further management.

## Introduction

Gastric cancer is the fifth most common cancer worldwide and remains one of the most deadly diseases, with a poor prognosis that often presents complex challenges in management, particularly in cases of distant metastasis [1]. While the liver and peritoneum are commonly affected sites, colon metastasis is a rare and unique phenomenon. On the other hand, gastric and colon cancer can occur simultaneously, for which differentiating between simultaneous primaries and metastatic disease is imperative in managing these patients [2]. Mutation in the E-cadherin (CDH1) suppressor gene is associated with an aggressive form of disease known as hereditary diffuse gastric cancer (HDGC) and is seen in 1-3% of cases, which is further complicated by the fact that patients typically remain asymptomatic until upon presentation [3]. Although surgery remains pivotal in the management of early-stage gastric cancer, addressing metastasis generally demands a comprehensive strategy integrating chemotherapy. We present a unique case of diffuse hereditary gastric cancer metastasizing to the colon in a young male being evaluated for persistent mild abdominal pain and iron deficiency anemia, highlighting the complexities of diagnosis and management in such atypical presentations.

## Case presentation

This is the case of a 35-year-old Hispanic male with a past history of *Helicobacter pylori* infection who was referred to the emergency department by his primary care physician (PCP) for evaluation of microcytic anemia. The patient initially presented to his PCP with a three-month history of persistent, dull epigastric pain that radiated to the back. Although pain was not worsened by food intake, he experienced associated early satiety, intermittent nausea, and a secondary 20 lb weight loss during the same period. During outpatient workup and management, the patient was treated for a positive *H. pylori* breath test and was noted to have progressively worsening anemia, prompting a referral to the emergency department for evaluation. The patient denied overt gastrointestinal (GI) bleeding, toxic habits, non-steroidal anti-inflammatory drug use, or family history of GI malignancy. Upon further review of systems, he reported mild exertional dyspnea but denied chest pain, dizziness, lightheadedness, night sweats, fever, or chills. Physical examination revealed a chronically ill-appearing young male with mild temporal wasting suggestive of malnutrition, with scleral and skin pallor and a soft, nondistended abdomen with tenderness in the right lower quadrant and left lower quadrant on deep palpation. 

Subsequent laboratory workup revealed abnormalities, including a decreased hemoglobin level, an increased platelet count, and a decreased ferritin and albumin level, otherwise grossly unremarkable (Table 1).

**Table 1:** Laboratory investigations on presentation. CEA: carcinoembryonic antigen; AST: aspartate transaminase; ALT: alanine transaminase; ALP: alkaline phosphatase.

Blood chemistry	Value	Reference range
White blood cell	5.7	4.40-11.0 x 10^3^/uL
Hemoglobin	8.1	13.5-17.5 g/dL
Hematocrit	24.9	38.8-50.0%
Platelets	460	150-450 x 10^3^/uL
Ferritin	3.7	24.0-336.0 ng/mL
CEA	<2	0-3.0 ng/mL
Sodium	142	136-145 mmol/L
Chloride	107	90-110 mmol/L
AST	21	10-36 U/L
ALT	17	6-46 U/L
ALP	75	33-130 U/L
Total bilirubin	0.3	0.2-1.2 mg/dL
Total protein	6.2	6.4-8.3 g/dL
Albumin	3.8	3.6-5.1 g/dL

**Figure 1:**
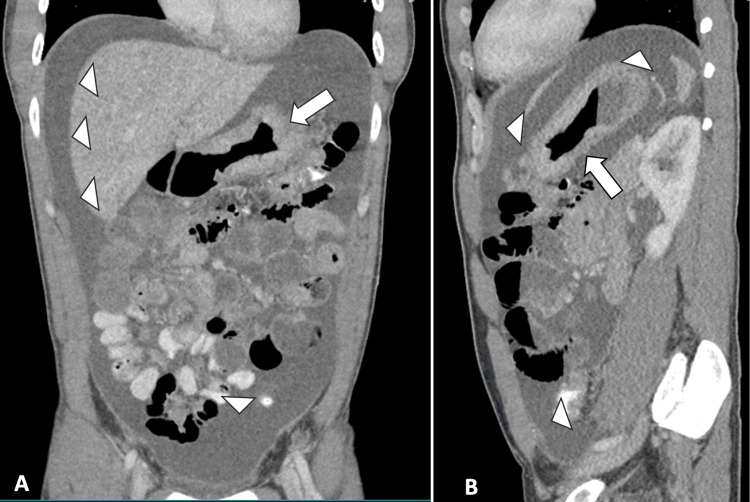
CT scan of the abdomen and pelvis with oral and intravenous contrast. (A) Coronal and (B) sagittal views showing gastric thickening (arrow) and diffuse ascites (arrowheads). CT: computed tomography.

Computed tomography (CT) scan of the chest, abdomen, and pelvis with oral and intravenous contrast revealed an abnormal stomach appearance suspicious for a circumferential gastric mass, indicative of linitis plastica as well as ascites with peritoneal thickening and scattered areas of peritoneal nodularity. No suspicious thoracic cavity lesions were present (Figure 1).

**Figure 2:**
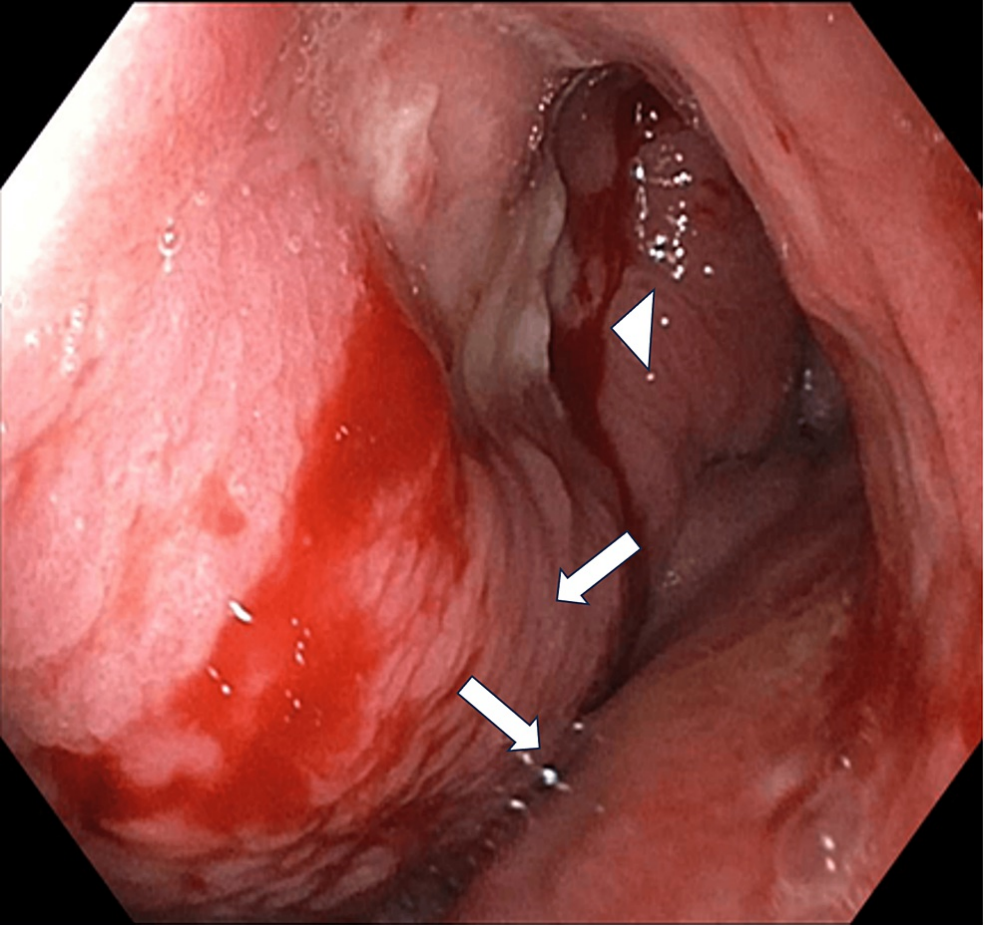
Esophagogastroduodenoscopy. Endoscopic gastric image of gastric body approximating antrum showing diffuse thickening of gastric mucosa (arrows) with ulcerated area and spontaneous bleeding (arrowhead).

**Figure 3:**
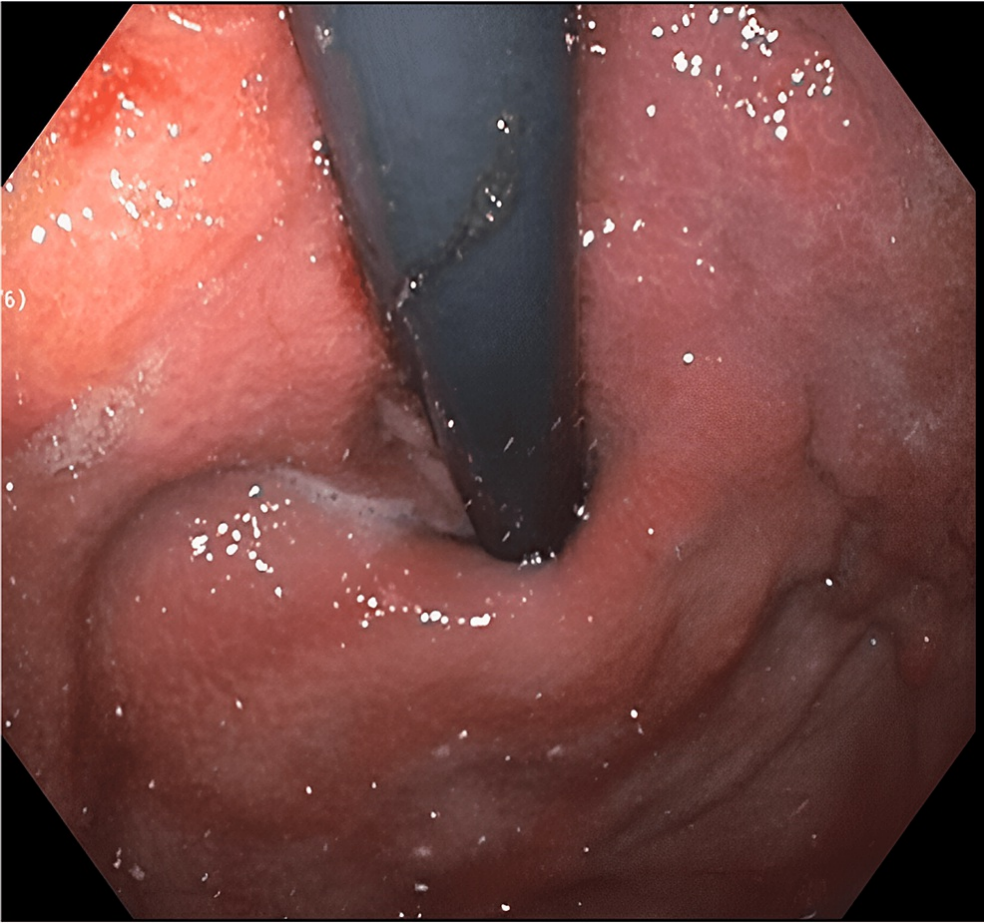
Esophagogastroduodenoscopy. Retroflexed view of gastric fundus and cardia showing diffusely thickened mucosa.

Esophagogastroduodenoscopy revealed a diffuse infiltrative circumferential mass with an area of ulceration with spontaneous bleeding extending from the fundus/cardia to the gastric body and antrum (Figures 2, 3).

**Figure 4:**
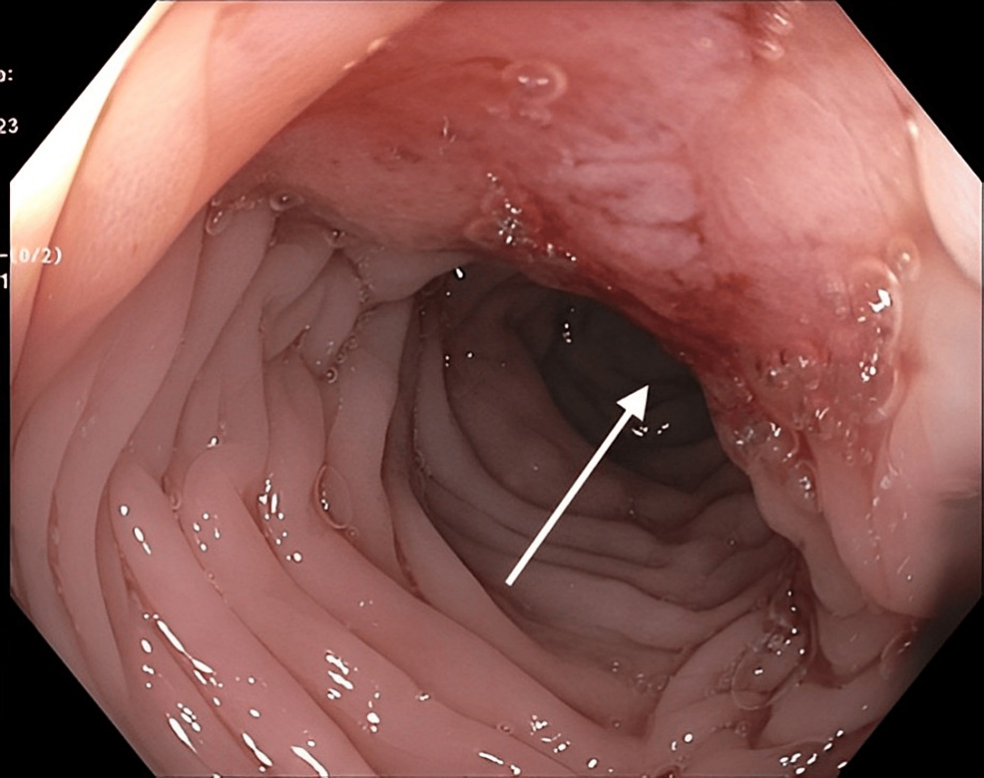
Endoscopic view of sigmoid colon showing a 20 mm lesion (arrow), Paris classification mixed IIa + IIc (flat elevation + mucosal depression).

**Figure 5:**
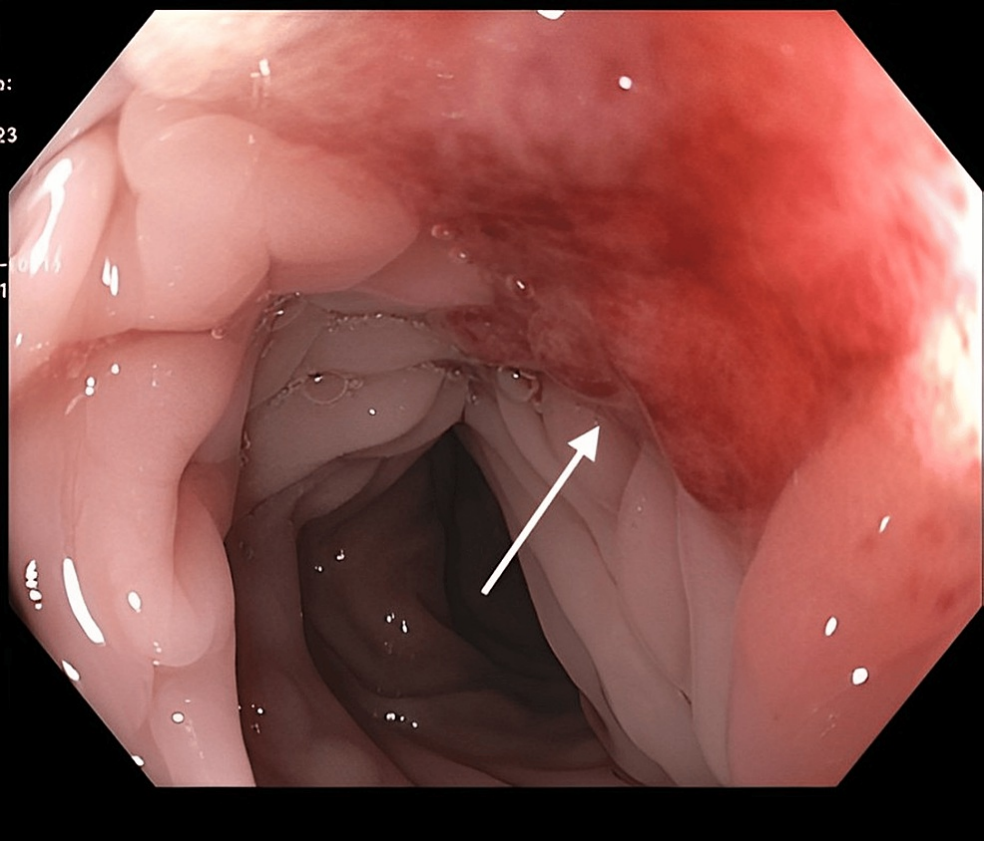
Endoscopic view of sigmoid colon showing a 20 mm lesion (arrow), Paris classification mixed IIa + IIc (flat elevation + mucosal depression), 20 cm from the anal verge.

Although a colonoscopy was attempted, the procedure was aborted due to significant resistance encountered while advancing the colonoscope through the sigmoid colon. Nonetheless, a 20 mm lesion, Paris classification mixed IIa + IIc, was discovered in the sigmoid colon around 20 cm from the anal verge (Figures 4, 5).

**Figure 6:**
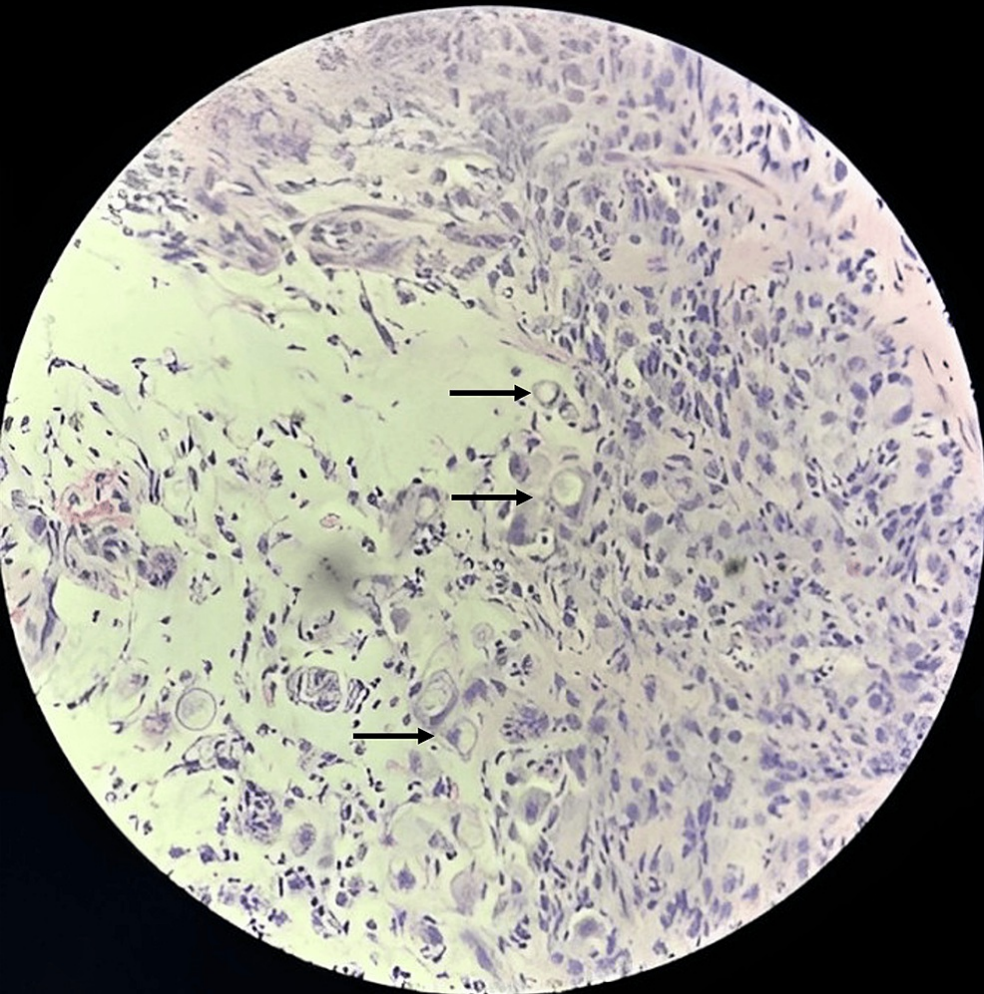
Gastric biopsies. Hematoxylin and eosin staining revealed a gastric mass of infiltrative, poorly differentiated adenocarcinoma with signet ring cells (arrows) features poorly cohesive diffuse subtype.

Gastric biopsies with hematoxylin and eosin staining revealed a gastric mass of infiltrative, poorly differentiated adenocarcinoma with signet ring features poorly cohesive diffuse subtype (Figure 6).

**Figure 7:**
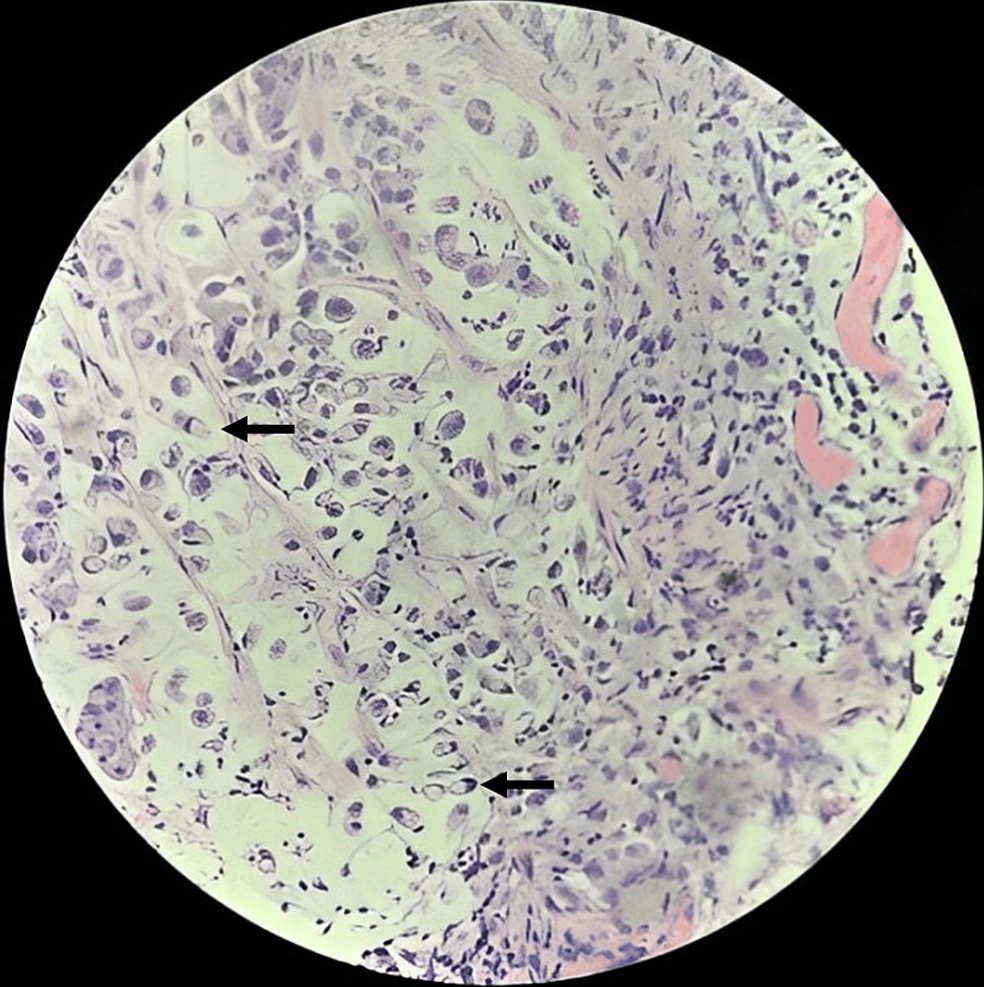
Colonic lesion biopsies. Hematoxylin and eosin staining revealed metastatic adenocarcinoma with signet ring cells (arrows).

Sigmoid colon lesion biopsies exhibited metastatic adenocarcinoma with a similar morphology to gastric biopsies, including signet ring cells (Figure 7).

In addition, immunohistochemistry showed that these tumor cells were positive for cytokeratin (CK) 7 and negative for CK20 and CDX2, suggestive of gastric origin. CD20 demonstrated a few positive cells. KRAS testing yielded negative results. HER2 immunohistochemistry resulted negative, with programmed death-ligand 1 (PDL1) combined positive score (CPS) of 15%. There was no loss of mismatch repair (MMR) and no evidence of microsatellite instability-high (MSI-H). Next-generation sequencing (NGS) studies were conducted, revealing a CDH-1 mutation confirming a diagnosis of familial gastric cancer. Palliative chemotherapy with six to eight cycles of FOLFOX was initiated, with a plan to add nivolumab in the third cycle of treatment. Repeat imaging was scheduled after four to six months of palliative chemotherapy to assess treatment efficacy. 

## Discussion

Gastric cancer is the fifth most commonly diagnosed malignancy worldwide, with over 1 million new cases annually, and the third most common cause of cancer-related deaths due to diagnosis at later stages of the disease. The incidence is two times higher in males than females, with a higher prevalence in East Asia, Eastern Europe, and South America [1]. While a wide array of risk factors contribute to the development of gastric cancer, *H. pylori* infection, along with dietary factors such as nitrated and salted food, is known to be the leading cause [4]. 

Mutation in the *CDH1* suppressor gene is associated with HDGC, seen in 1-3% of cases [2]. This low prevalence underscores the rarity of this mutation, placing our patient within this distinctive subset. Dysregulation of CDH1 leads to tumor proliferation, invasion, migration, and metastasis. This mutation follows an autosomal dominant mode of inheritance in HDGC, with mutated alleles of CDH1 present in 30-50% of cases [3]. Furthermore, CDH1 mutations are most commonly associated with invasive lobular breast cancer, which accounts for 5-15% of sporadic breast cancers compared to 85-95% of ductal carcinoma associated with BRCA1/2 mutations. A recent study of 75 families with CDH1 mutations found that the lifetime risk for developing diffuse gastric cancer or lobular breast carcinoma for men is 70% and for women 56% [5]. These data underscore the critical importance of close management for carriers of CDH1 mutations, given the aggressive nature of this mutation and poor prognosis when advanced. One of the challenges in managing CDH1 mutations is that patients typically remain asymptomatic upon presentation [3]. As seen in our patient's case, symptoms only manifest once the cancer has metastasized, complicating early detection and intervention efforts. This highlights the need for proactive surveillance and screening protocols within affected families to detect and address potential malignancies at the earliest possible stage.

Metastatic spread of gastric cancer has become more prevalent in recent years, with cancers typically spreading to the liver, peritoneum, and lymph nodes through various mechanisms. Common routes of metastasis include lymphatics, blood, and through the peritoneum [6]. Metastasis in the colon accounts for 1% of total colorectal cancers, primarily from the lung and ovary and less commonly from the mammary gland, prostate, skin, and kidney [7]. This rarity can be explained by the separate blood supply and lymphatics between the stomach and colon. Moreover, the colon possesses a distinct environment less favorable for gastric cell survival, thereby limiting metastatic tumor formation. Despite this, recent years have seen a rise in reports of gastric cancer metastasizing to the colon, often observed in patients over the age of 40 [6-11]. Consideration of these factors helps to highlight the uniqueness of this patient's case. Not only is our patient below the average age of documented cases, but he also possesses a rare mutation of gastric cancer and an uncommon metastasis. These distinctive features underscore the need for an individualized treatment plan, which can only be devised through a comprehensive understanding of the histopathology and molecular mechanisms underlying the cancer. 

Gastric adenocarcinoma is classified into five histologic subtypes (Table 2) [12]. 

**Table 2:** Histologic subtypes.

Histologic subtypes	
Tubular adenocarcinoma	Most common subtype generally associated with early gastric carcinoma
Papillary adenocarcinoma	Present mostly in older people and is frequently associated with lymph node involvement and liver metastasis
Mucinous carcinoma	Accounts for only 10% of cases
Signet ring cell carcinoma	Tends to present with lymphovascular vascular invasion and lymph node metastasis with a predisposition to invade duodenum via submucosal and subserosal routes
Uncommon variants	Include adenosquamous carcinoma, choriocarcinoma, and undifferentiated carcinoma, among others

Lauren’s criteria further classify gastric adenocarcinoma into intestinal, diffuse, and indeterminate subtypes. The intestinal subtype accounts for 54% of cases and is associated with *H. pylori* infection and intestinal metaplasia; the diffuse subtype represents 32% and is often seen in young females, and the remaining 15% is of the indeterminate subtype, which is an uncommon variant. Bormann’s classification is based on the gross appearance of gastric carcinomas, divided into four types. Type I polypoid carcinoma, type II fungating carcinoma, type III ulcerated carcinoma, and type IV diffusely infiltrative carcinoma, known as linitis plastica in signet cell carcinoma when the majority of the gastric wall is involved by an infiltrating tumor [12].

CT scans are commonly used for initial evaluation, revealing wall thickening, nodularity, and perigastric lymphadenopathy. Although CT scans may provide greater insight into the extent of metastasis, they have a low diagnostic value [8]. PET scans provide functional information about tumor metabolism, aiding in detecting distant metastases and assessing treatment response [13]. In addition to conventional radiological imaging modalities, endoscopic ultrasound (EUS) plays a significant role in the imaging and staging of gastric cancer. EUS offers high-resolution imaging of the GI wall layers and adjacent structures, providing information about tumor size, depth of invasion, and involvement of regional lymph nodes. By combining endoscopic visualization with ultrasound imaging, EUS allows for precise localization of lesions, assessment of tumor morphology, and accurate determination of tumor stage [14].

Treatment for HDGC is based on disease extent; in localized disease, surgical resection with neoadjuvant or adjuvant chemotherapy and radiation therapy is standard. However, metastatic disease treatment typically involves chemotherapy and immunotherapy alone, focusing on providing palliative treatment by aiding in symptom management and life extension. Immune checkpoint blockade therapy, targeting PD-L1 and CTLA-4, has emerged as a viable treatment option in the past decade. Pembrolizumab, in combination with trastuzumab, is recommended as the first line for locally advanced, unresectable, and metastatic HER-2-positive gastric carcinoma. This therapy has also been shown to be beneficial in treating recurrent cases with PDL-1 expression.

For HER-2-negative cases with increased PDL-1 expression, such as our patient, nivolumab combined with chemotherapy is recommended [3]. NCCN guidelines endorse various chemotherapy regimens, such as fluoropyrimidines (fluorouracil or capecitabine) with oxaliplatin and trastuzumab, or cisplatin and trastuzumab, often with pembrolizumab based on PD-L1 status. Second-line therapies include fluorouracil and irinotecan, paclitaxel with or without cisplatin, docetaxel with or without cisplatin, and various combinations of these drugs. The most commonly used HER2-negative regimens typically include FOLFOX (folinic acid, fluorouracil [5-FU], and oxaliplatin) or CAPOX (capecitabine and oxaliplatin) [15]. Alternatively, fluorouracil with cisplatin may be used, sometimes augmented with pembrolizumab. In our patient, who had an aggressive form of hereditary diffuse cell gastric carcinoma with metastasis to the colon, FOLFOX with nivolumab was selected as the appropriate chemotherapy regimen for palliative intent. 

Families of patients affected by HDGC not only face the burden brought on by the diagnoses of their affected family members but also face the daunting uncertainty of their own potential risk of the disease. Genetic screening is recommended for patients who have a familial history of two or more cases of gastric cancer, with at least one being diffuse cancer. Patients who have a history of lobular breast cancer in the family may also qualify for genetic testing, given that both lobular breast cancer and HDGC are associated with the same CDH1 mutation. For family members who test negative for the mutation, recent recommendations suggest yearly endoscopic surveillance for up to two years following the negative test [16]. It should be taken into account that there are uncertainties regarding endoscopic surveillance, as negative results may offer a false sense of reassurance and may be used to justify delaying gastrectomy [3].

Given the increased risk of gastric cancer associated with CDH1 mutation, ranging up to 70% in men and 56% in women, appropriate screening measures should be taken into consideration in a timely manner [17]. For asymptomatic carriers of CDH1 mutation who also possess a blood relative with HDGC, total prophylactic gastrectomy (PTG) may be considered, with or without pouch reconstruction. This recommendation is supported by one study that reported evidence of positive postoperative pathology despite negative preoperative endoscopies [3]. Nevertheless, the decision to undergo PTG carries substantial physical and psychological implications that should not be overlooked. Patients considering PTG should be evaluated thoroughly to address potential long-term consequences, including immune, pharmacokinetic, and psychological impacts [5]. 

## Conclusions

HDGC is associated with *CHD1* gene mutation and manifests as an aggressive form of cancer that typically presents as a poorly differentiated lesion, often metastatic at presentation. However, colonic metastasis remains a rare occurrence that should be promptly recognized and that often presents significant management challenges, especially in younger patients. While surgery with or without chemotherapy remains the only curative option for early gastric cancer, immunotherapy with checkpoint inhibitors and chemotherapy remain possible options for patients with advanced disease. Given the aggressive nature of this disease and its potential for hereditary transmission, family members of patients with HDGC should consider proactive measures such as genetic counseling, testing, and regular surveillance endoscopies in order to intervene at the earliest possible stage and improve outcomes. Regarding colonic metastasis from gastric cancer, further research is warranted to understand the underlying molecular mechanisms of this rare metastasis, thereby improving diagnostic accuracy and early detection.
